# Neuroprotective effects of goji berry (*Lycium barbarum* *L.*) polysaccharides on depression-like behavior in ovariectomized rats: behavioral and biochemical evidence

**DOI:** 10.3325/cmj.2023.64.231

**Published:** 2023-08

**Authors:** Hayriye Soytürk, Bihter Gökçe Bozat, Fatma Pehlivan Karakas, Hamit Coskun, Tulin Firat

**Affiliations:** 1 Department of Interdisciplinary Neuroscience, Health Sciences Institute, Bolu Abant Izzet Baysal University, Bolu, Turkey; 2 Department of Biology, Faculty of Science and Art, Bolu Abant Izzet Baysal University, Bolu, Turkey; 3 Department of Psychology, Faculty of Science and Art, Bolu Abant Izzet Baysal University, Bolu, Turkey; 4 Department of Histology and Embryology, Faculty of Medicine Bolu Abant Izzet Baysal University, Bolu, Turkey

## Abstract

**Aim:**

To assess the protective effects of goji berry (*Lycium barbarum* L.) polysaccharides (LBP) on depression-like behavior in ovariectomized rats and to elucidate the mechanisms underlying these effects.

**Methods:**

One hundred female Wistar albino rats (three months old) were randomly assigned either to ovariectomy (n = 50) or sham surgery (n = 50). After a 14-day recovery period, the groups were divided into five treatment subgroups (10 per group): high-dose LBP (200 mg/kg), low-dose LBP (20 mg/kg), imipramine (IMP, 2.5 mg/kg), 17-beta estradiol (E2, 1 mg/kg), and distilled water. Then, rats underwent a forced swimming test. We also determined the levels of serum antioxidant enzymes (superoxide dismutase, catalase, glutathione peroxidase, and malondialdehyde), E2 levels, hippocampal brain-derived neurotrophic factor (BDNF), 5HT2A receptor, and transferase dUTP nick end labeling (TUNEL)-positive cells.

**Results:**

Both low-dose LBP and imipramine decreased depression-like behavior by increasing serum superoxide dismutase activity and by decreasing serum malondialdehyde level. Furthermore, low-dose LPB, high-dose LBP, and imipramine increased the number of 5-HT2A receptor- and BDNF-positive cells but decreased the number of TUNEL-positive cells in the hippocampus.

**Conclusion:**

This is the first study to show the antidepressant effect of LBP. Although additional research is needed, LBP may be considered a potential new antidepressant.

Depression is one of the most serious health issues (1), affecting women twice as often as men (2). A factor contributing to the development of depression is an increase in oxidative stress in the brain (3). Oxidative stress builds up as a result of irregular formation and removal of reactive oxygen species (ROS) by the antioxidant defense system (4).

Antioxidant enzyme activities in patients with depression are lower than in healthy individuals (5,6). Oxidative stress negatively affects cellular metabolism. Studies on lipid peroxidation in patients with depression report increased malondialdehyde (MDA) (7,8) and other lipid peroxidation products (9). Oxidative stress has also been associated with cell damage (7), apoptosis (10), and terminal deoxynucleotidyl transferase dUTP nick end labeling (TUNEL)-positive status. Oxidative stress is also related to decreased estrogen levels (9). This results in changes in neuromodulators and neurotransmitters such as brain-derived neurotrophic factor (BDNF) (11,12) and serotonin (5-HT) in the hippocampus (13-15). Furthermore, depression causes a decrease in hippocampal volume (16).

17-beta estradiol (E2), a form of estrogen, gradually decreases during the postmenopausal period. The decrease in the E2 level disrupts behavior by alterations in brain plasticity, neurogenesis, neurotransmitters, oxidative stress, inflammation, and apoptosis (17). The administration of E2 to ovariectomized rats decreases depressive-like behavior (18).

Menopausal depression is treated with selective 5-HT reuptake inhibitors, 5-HT and noradrenaline (NA) reuptake inhibitors, and NA and specific 5-HT reuptake inhibitors (1). One of the antidepressant drugs is imipramine (IMP), a tricyclic antidepressant (2) restoring decreased BDNF levels in the hippocampus (3,4). Another treatment method is estrogen replacement therapy (ERT) (7). ERT was found to ameliorate increased oxidative stress in ovariectomized rats (10). Endogenous estrogen affects depression by regulating dopamine, 5-HT, and NA metabolism in the prefrontal cortex. However, estrogen therapy to enhance the effectiveness of antidepressant treatment has not gained ground due to its potentially detrimental side effects or interactions with other drugs (19). Because of these undesirable effects, herbal-based alternative ERT treatments are used (20). These alternatives include phytoestrogens, vitamins, minerals, antioxidants, antidepressants, and memory enhancing medications (9,13). For instance, grape powder was found to prevent depression induced by oxidative stress (9). Due to its polysaccharide content, *L. barbarum* (goji berry) is a potent antioxidant fruit (16). LBP exerts neuroprotective effects by decreasing oxidative stress and apoptosis (11). In a recent study, we revealed positive benefits of *L. barbarum* methanol extract on rat behavior (12). We also showed that LBP decreased anxiety-like behavior of ovariectomized rats (14). LBP has neuroprotective properties such as reducing caspase-3 activation and apoptosis level in the hippocampus (15). As a result, LBP may be a promising candidate for the treatment of neuronal apoptosis-induced depression (21).

Even though goji berry LBP have anti-inflammatory, antioxidant, and anti-aging effects, the neuroprotective effects of LBP on depression induced by decreased levels of estrogen have not been studied so far. Furthermore, LPB in different doses has not been compared with a positive control, including current pharmaceutic medicines. Therefore, the aim of this study was to assess how different LPB doses affect superoxide dismutase (SOD), catalase (CAT), glutathione peroxidase (GPX), and malondialdehyde (MDA) levels (6), as well as the hippocampal levels of BDNF, 5-HT2AR, and TUNEL-positive cells in an animal menopausal depression model with suitable control groups.

## MATERIAL AND METHODS

### Animals

Female Wistar albino rats (90 days old) were purchased from the Department of Experimental Animal Center, Bolu Abant Izzet Baysal University. Individual rats were housed in plastic cages (40 × 50 × 20 cm) at a temperature of 22 °C under 12/12 h light/dark cycles. Until the trials began, food and drink were provided *ad libitum*. To avoid potential circadian disruption, all experiments were carried out between 13:00 and 17:00 h. The animals did not eat for 12 hours before gavage. The study was approved by the Institutional Ethics Committee for Animal Research of Bolu Abant Izzet Baysal University (23076.046121/2011-81).

### Experimental groups

A total of 100 female rats were randomly allocated to either the ovariectomy (OVX, n = 50) or sham surgery group (SHAM, n = 50). Ovariectomy was performed by making an incision (2-cm long) along the linea alba. The fallopian tubes and ovaries were cauterized, crushed, and light-cauterized, and then bilaterally removed by cutting above the clamped area. The rats in the sham-operated group were exposed to the same OVX process except for the ovary removal.

Following a 14-day recovery period, treatments were administered through gavage (3 mL/kg) for 20 days in a row. Stock solutions were made by dissolving the substances in distilled water (DW). Both surgical groups were divided into five therapy subgroups (n = 10): high-LBP (HD-LBP, 200 mg/kg); low-LBP (LD-LBP, 20 mg/kg); IMP (2.5 mg/kg); 17 beta-estradiol (E2, 1 mg/kg); and (E) DW.

### Preparation of L. barbarum polysaccharides

L. *barbarum* fruits were obtained from GojiForm (Sivas, Turkey). Two hundred grams of dried fruits were subjected to 600 mL of chloroform: methanol (2:1 v/v) for two times, 600 mL of 80% ethanol for two times, and to hot water (80 °C) for three times, in that order. The reflux was extracted with 95% ethanol for precipitation at +4 °C. The precipitate was centrifuged and rinsed with pure ethanol and acetone, then centrifuged and washed again. It was then dried under low pressure. HD -LBP and LD-LPB doses were made.

### Drugs

E2 was purchased from Sigma-Aldrich (St. Louis, MO, USA). Stock solutions were created by dissolving E2 in 0.1 mL of 100% ethanol and adding it to DW. The final ethanol concentration was decreased to 1%. This dose was found to be safe and effective for inducing uterotrophic responses in ovariectomized subjects (22). IMP (Sigma-Aldrich) was dissolved in DW and administered orally through gavage.

### Forced swimming test

After all treatment protocols were completed, the rats underwent a forced swimming test (FST), a method often used to detect immobility and depression-like behavior. Individual rats were placed separately in a glass cylinder (diameter 24 cm; height 53 cm, water depth 40 cm) filled with water (temperature of 26-28 °C). After a 15-minute training session in the water, the rats were returned to their cages. The animals were placed in the water for a 5-minute session the next day. The entire period of immobility (floating) throughout the test session was recorded with the EthoVision video tracking system, version 6 (Noldus Ethovision, Wageningen, Netherlands; Commat LTD, Ankara, Turkey). The result was expressed in minutes.

### Blood sampling and biochemical assays

After the behavioral testing, blood samples were obtained. The samples were centrifuged to obtain serum for the determination of E2 and oxidative stress biomarkers: SOD, CAT, GPX, and MDA with the Sunred ELISA kit (Sunred, Shanghai, China). The procedure was carried out according to the manufacturer’s protocol (23).

### Tissue collection and immunohistochemical analysis

Following the FST, five rats from each group were randomly selected for histological tests. After anesthesia with a ketamine-xylazine overdose, perfusion fixation was used. The brain tissues were extracted and put in a 10% neutral formaldehyde solution. After a 72-hour fixation, right lobes were subjected to the analysis. The number of 5-HT2AR, BDNF, and TUNEL-positive cells in the hippocampus tissue was determined with immunohistochemical staining and TUNEL staining procedures according to the manufacturer’s protocol (Millipore, Burlington, MA, USA).

Immunohistochemistry SER and BDNF staining was identified as either negative or positive. Positive staining was defined as the presence of brown chromogen on the edge of the hematoxylin-stained cell nucleus, within the cytoplasm, or in the membrane. Photos were taken with a digital camera (Canon, Tokyo, Japan) at 20 × , 40 × , and 63.5 × magnification.

### Statistical analysis

The normality of distribution was tested with the Shapiro Wilk test. The data are presented as mean ± standard deviation. The differences between the groups undergoing two procedures (SHAM and OVX) and five treatments (HD-LBP, LD-LBP, IMP, E2, and DW) were assessed with a two-way ANOVA and a Bonferroni test. A *P* value of 0.05 was considered significant. The analysis was conducted with IBM SPSS, 19.0 for Windows (IBM Corp., Armonk, NY, USA).

## RESULTS

### Immobility duration

**Figure 1:**
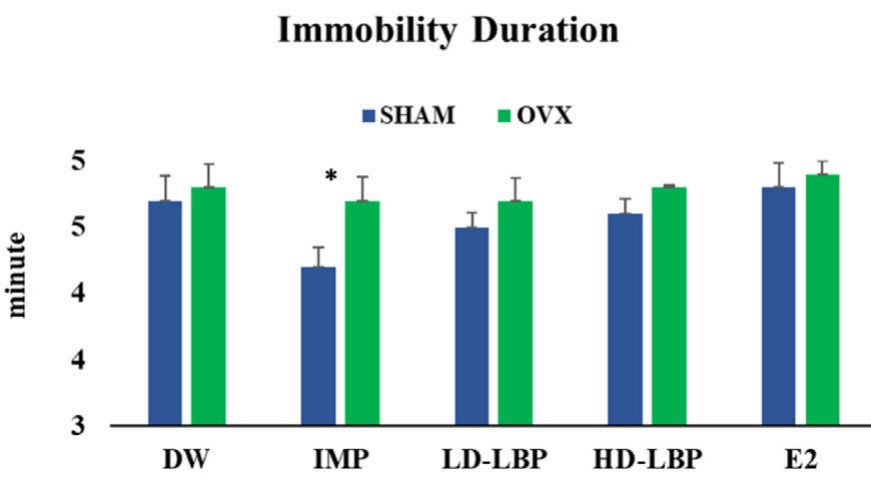
Effect of high dose of *Lycium barbarum* L. polysaccharides (HD-LBP), low dose of LBP (LD-LBP), imipramine (IMP), 17 beta estradiol (E2), and distilled water (DW) on the immobility time in ovariectomized (OVX) and sham-operated rats. Bars represent the mean ± standard deviation of immobility duration on the forced swimming test. *IMP vs HD-LBP, LD-LBP, and DW, *P* < 0.05; and IMP-SHAM vs IMP OVX, *P* < 0.05 (two-way ANOVA-Bonferroni test).

The length of immobility on FST was significantly affected by treatment (F [4, 84] = 20.85, *P* = 0.0001, η^2^ = 0.50). The IMP groups were less immobile than the HD-LBP, E2, and DW groups (M_IMP_ = 4.45<M_HD-LBP_ = 4.72<M_DW_ = 4.77<M_E2_ = 4.88). The length of immobility on FST was also significantly affected by the procedure (F [1, 84] = 41.31, *P* = 0.0001, η^2^ = 0.33). The SHAM groups were less immobile than the OVX groups (4.59 vs 4.79). There was a substantial interaction impact between therapy and surgery (F [4, 84] = 3.99, *P* = 0.005, η^2^ = 0.16). The IMP-SHAM group was less immobile than the IMP-OVX group (4.23 vs 4.68) (Figure 1).

### SOD concentration

**Figure 2:**
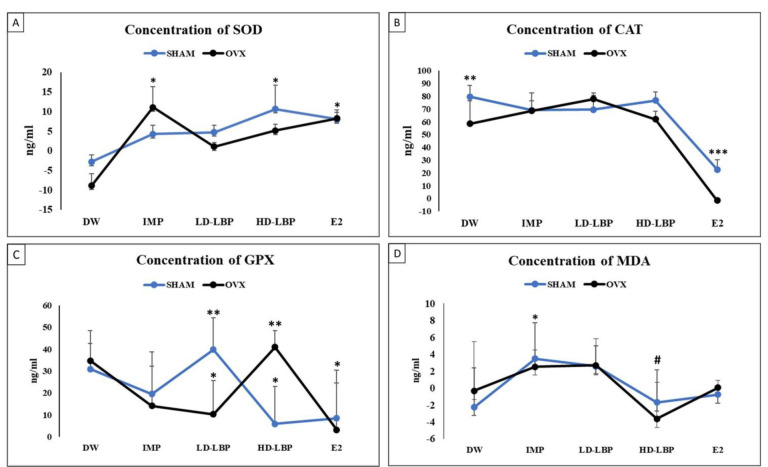
Effect of high dose of *Lycium barbarum* L. polysaccharides (HD-LBP), low dose of LBP (LD-LBP), imipramine (IMP), 17 beta estradiol (E2), and distilled water (DW) on the serum level of superoxide dismutase (SOD) (**A**), catalase (CAT) (**B**), glutathione peroxidase (GPX) (**C**), and malondialdehyde (MDA) (**D**) in ovariectomized (OVX) and sham-operated rats. Bars represent the mean ± standard deviation of these biochemical parameters. * *P* < 0.05 compared with DW groups; ** *P* < 0.05 compared within groups (OVX vs SHAM); *** *P* < 0.05 compared with both DW groups and compared within groups (OVX vs SHAM); # *P* < 0.05 compared with the LD-LBP and IMP treatment groups (two-way ANOVA-Bonferroni test).

SOD concentration was significantly affected by treatment (F [4, 47] = 4.16, *P* = 0.006, η^2^ = 0.26). The HD-LBP, E2, and IMP groups demonstrated greater SOD levels than the DW groups (M_E2_ = 8.10>M_HD-LBP_ = 7.86>M_IMP_ = 7.63>M_DW_ = -5.85). SOD concentration was also significantly affected by the procedure (F [4, 47] = 0.95, *P* > 0.44). The DW-SHAM group had a higher SOD concentration than the DW-OVX group (-2.85 vs -8.87) and the IMP-SHAM group had a lower SOD concentration than the IMP-OVX group (4.24 vs 11.02). The E2-SHAM group had a similar concentration as the E2-OVX group (M_E2-SHAM_ = 8.00, M_E2-OVX_ = 8.20) (Figure 2A).

### CAT concentration

CAT concentration was significantly affected by treatment (F [4, 48] = 27.30, *P* = 0.0001, η^2^ = 0.69). It was lower in the E2 groups than in the other groups (M_E2_ = 10.61<M_IMP_ = 68.83<M_DW_ = 69.16<M_HD-LBP_ = 69.33<M_LD-LBP_ = 73.71). CAT concentration was also significantly affected by the procedure (F [1, 48] = 5.00, *P* = 0.03, η^2^ = 0.09). It was higher in the SHAM groups than in the OVX groups (63.56 vs 53.10). Despite the fact that no significant interaction between therapy and procedure was found (F [4, 48] = 1.79, *P* > 0.05), CAT concentrations in the LD-LBP-SHAM and IMP-SHAM groups were similar to those in the OVX groups (M_LD-LBP-OVX_ = 77.91, M_LD-LBP-SHAM_ = 69.51, M_IMP-SHAM_ = 69.13, M_IMP-OVX_ = 68.53) (Figure 2B).

### GPX concentration

GPX concentration was significantly affected by treatment (F [4, 48] = 4.03, *P* = 0.007, η^2^ = 0.25). GPX level was lower in the E2 group than in the DW groups (5.81 vs 32.89). There was a substantial interaction between therapy and procedure (F [4, 48] = 4.91, *P* = 0.002, η^2^ = 0.29). GPX level was lower in the HD-LBP-SHAM than in the HD-LBP-OVX group (41.03 vs 5.91) and it was higher in the LD-LBP-SHAM group than in the LD-LBP-OVX (10.31 vs 39.83) (Figure 2C).

### MDA concentration

MDA concentration was significantly affected by treatment (F [4, 48] = 5.50, *P* = 0.001, η^2^ = 0.31). MDA levels were lower in the HD-LBP groups than in the LD-LBP groups (1.13 vs 1.08), and in the DW and HD-LBP groups than in the IMP groups (M_DW_ = -1.3<M_HD-LBP_ = 1.13<M_IMP_ = 2.99). There was no significant interaction between treatment and procedure (F [4, 48] = 0.48, *P* > 0.75). MDA levels were lower in the DW-SHAM group than in the DW-OVX group (-2.27 vs 0.33). In the LD-LBP-SHAM group, they were comparable with those in the LD-LBP-OVX group (M_HD-LBP-SHAM_ = -1.70, M_HD-LBP-OVX_ = -3.64) (Figure 2D).

### E2 concentration

**Figure 3:**
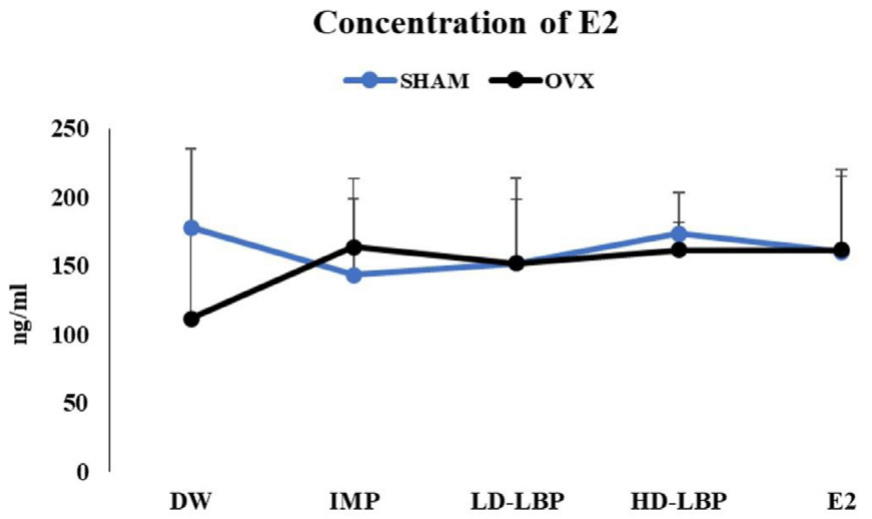
Effect of a high dose of *L. barbarum* polysaccharides (HD-LBP), low dose of LBP (LD-LBP), imipramine (IMP), 17 beta estradiol (E2), and distilled water (DW) on the serum level of E2 in ovariectomized (OVX) and sham-operated rats. Bars represents mean ± standard deviation of serum E2 levels (*P* > 0.05; two-way ANOVA-test).

E2 levels were not significantly affected by treatment (F [4, 48] = 0.28, *P* > 0.05) and there was no interaction effect between treatment and surgery (F [4, 48] = 1.10, *P* > 0.05). Although the DW-SHAM group had higher E2 levels than the DW-OVX group, this difference was not found in the other groups (M_DW-SHAM_ = 178.10>M_DW-OVX_ = 111.34; M_LD-LBP-SHAM_ = 151.93; M_LD-LBP-OVX_ = 151.94; M_E2-SHAM_ = 160.19; M_E2-OVX_ = 161.45) (Figure 3). The finding that serum E2 level in the DW-SHAM group was higher than that in the DW-OVX group confirms that the ovariectomy model was established.

### Histological results

**Figure 4:**
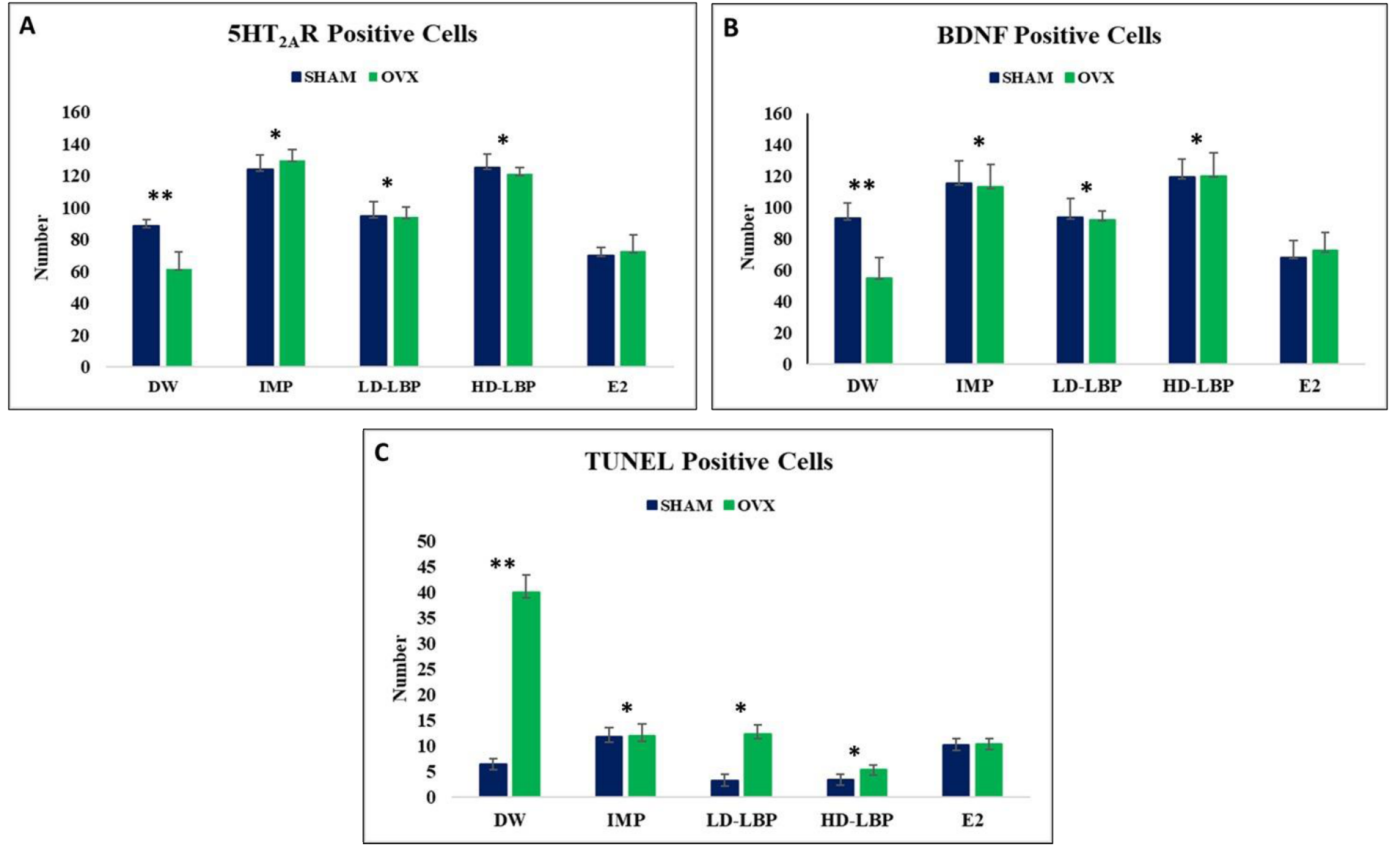
Effect of a high dose of *L. barbarum* polysaccharides (HD-LBP), low dose of *L. barbarum* polysaccharides (LD-LBP), imipramine (IMP), 17 beta estradiol (E2), and distilled water (DW) on hippocampal serotonin receptor 2A (5-HT2AR)-positive (**A**), brain derived neurotrophic factor (BDNF)-positive (**B**), and terminal deoxynucleotidyl transferase dUTP nick end labeling (TUNEL)-positive cells (**C**) of ovariectomized (OVX) and sham-operated groups. Bars represent mean ± standard deviation of these histochemical parameters. * *P* < 0.05 compared with the DW group; ** *P* < 0.05 compared within groups (OVX vs SHAM); *** *P* < 0.05 compared with both DW-treated groups and compared within groups (OVX vs SHAM) (two-way ANOVA-Bonferroni test).

**Figure 5:**
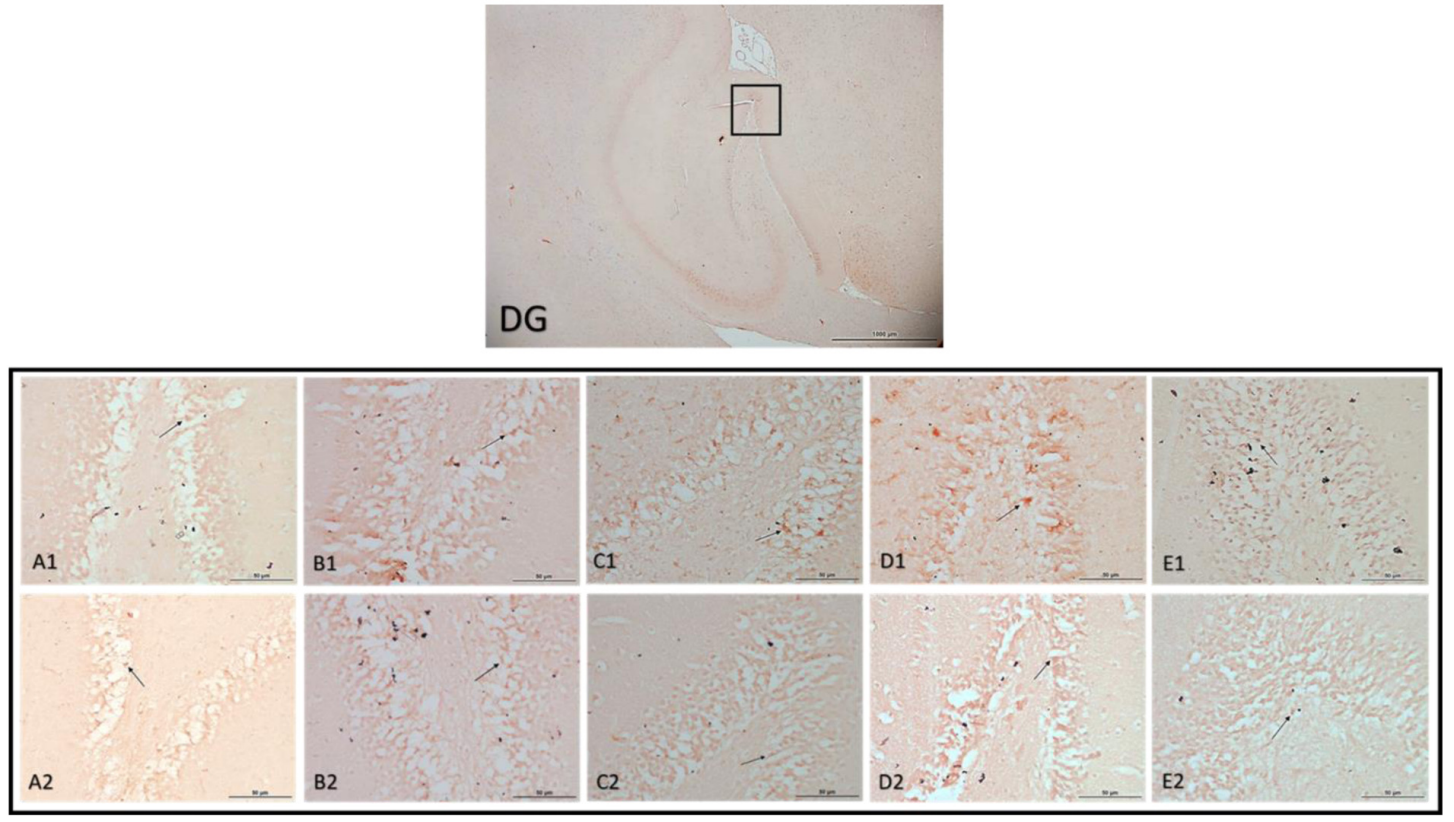
Effects of distilled water (**A**), 17-beta estradiol (**B**), low dose of *L. barbarum* (**C**), high dose of LBP (**D**), and imipramine (**E**) on immunostaining of hippocampal (dentate gyrus, DG, 2 × ) serotonin receptor 2A-positive cells in the sham (A-E1) and ovariectomy groups (A-E2) (20 × ).

**Figure 6:**
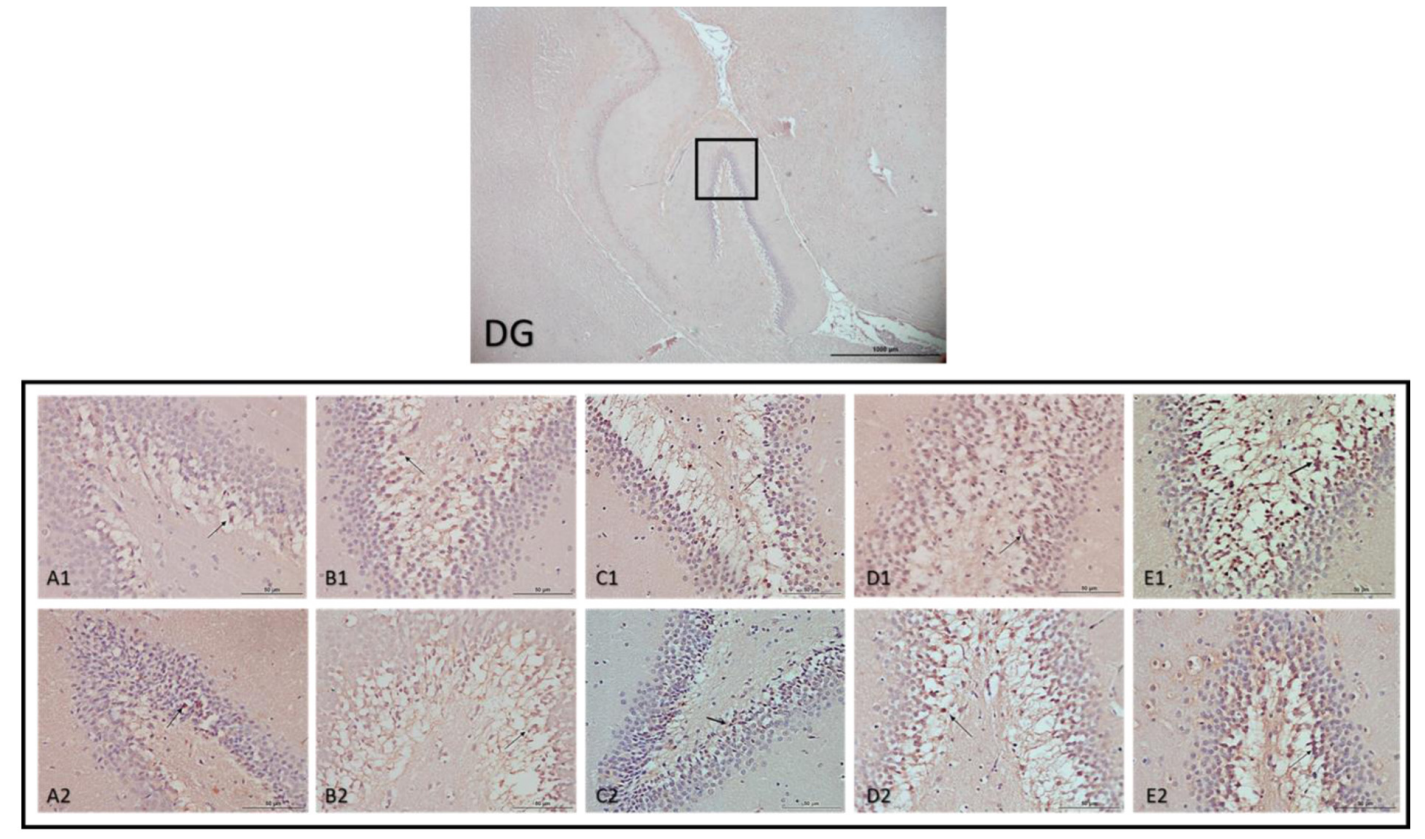
Effects of distilled water (**A**), 17-beta estradiol (**B**), low dose of *L. barbarum* (**C**), high dose of *L. barbarum* (**D**), and imipramine (**E**) on immunostaining of hippocampal (dentate gyrus, DG, 2 × ) brain derived neurotrophic factor-positive cells in the sham (A-E1) and ovariectomy groups (A-E2) (20 × ).

**Figure 7:**
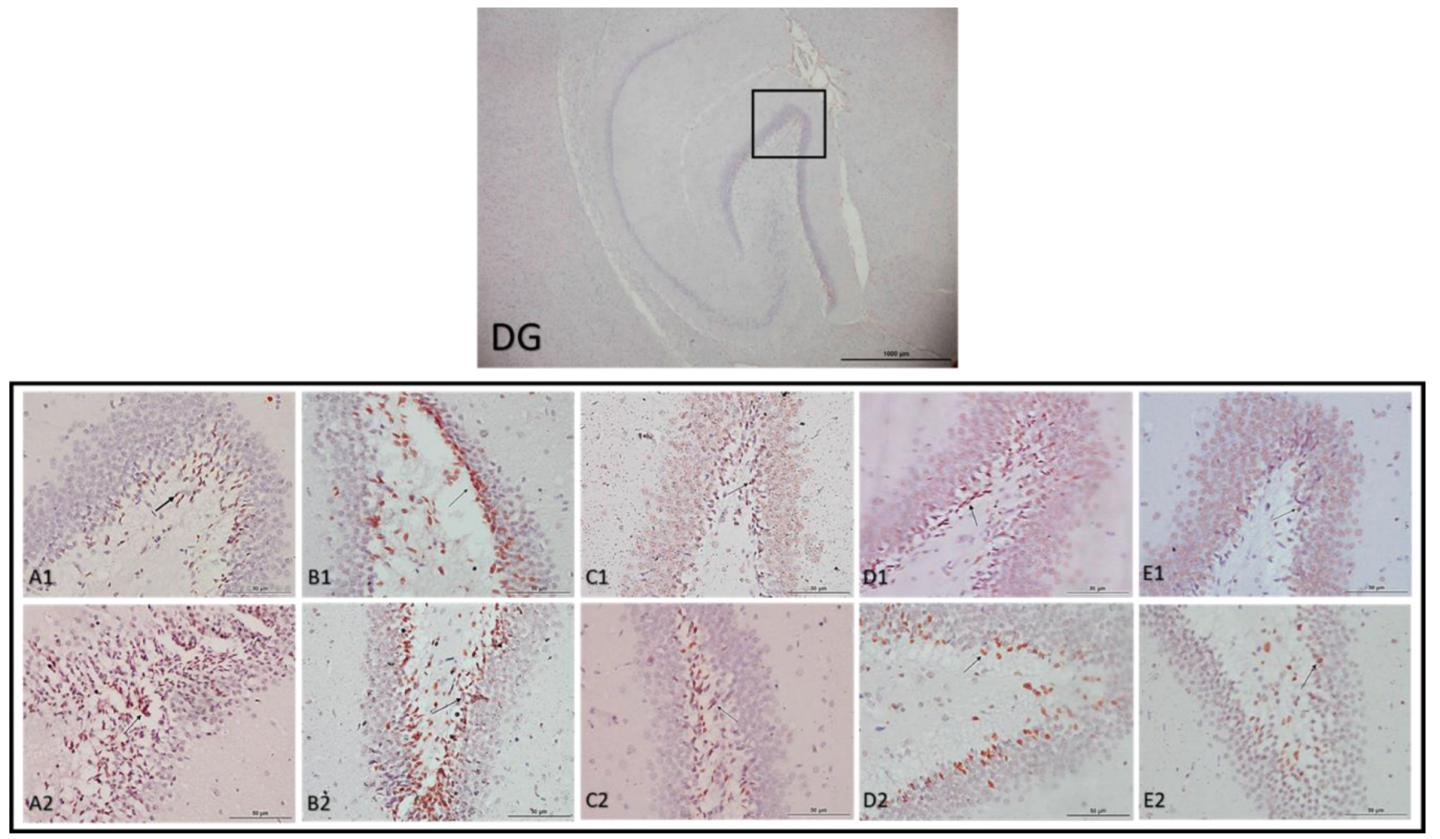
Effects of distilled water (**A**), 17-beta estradiol (**B**), low dose of *L. barbarum* (**C**), high dose of *L. barbarum* (**D**), and imipramine (**E**) on immunostaining of hippocampal (dentate gyrus, DG 2 × ) terminal deoxynucleotidyl transferase dUTP nick end labeling-positive cells in sham (A-E1) and ovariectomy groups (A-E2) (20 × ).

The number of 5-HT2AR, BDNF, and TUNEL-positive cells in the hippocampal region was significantly affected by treatment (F_5-HT2AR_ [4, 40] = 113.23, *P* = 0.001, η^2^ = 0.92; F_BDNF_ [4, 40] = 35.63, *P* = 0.001, η^2^ = 0.78; F_TUNEL_ [4, 40] = 162.85, *P* = 0.001, 2 = 0.94.1). 5-HT2AR and BDNF counts were higher and TUNEL-positive cell count was lower in the HD-LBP and IMP groups than in the other treatment groups. The number of positive cells in the hippocampal area was significantly differently affected by the procedure (F_5-HT2AR_ [1, 40] = 4.18, *P* = 0.048, η^2^ = 0.09; F_BDNF_ [1.40] = 4.62, *P* = 0.038, η2 = 0.10; F_TUNEL_ [1, 40] = 328.46, *P* = 0.001, η^2^ = 0.89). The SHAM groups had more 5-HT2AR and BDNF cells, and fewer TUNEL-positive cells, than the OVX groups. There was a substantial interaction effect between therapy and procedure (F _5-HT2AR_ [4, 40] = 7.02, *P* = 0.001, η^2^ = 0.41; F _BDNF_ [4, 40] = 5.33, *P* = 0.002, η^2^ = 0.35; F _TUNEL_ [4, 40] = 162.59, *P* = 0.001, η^2^ = 0.94) (Figure 4A-C). Among SHAM animals, the HD-LBP and IMP groups had more 5-HT2AR, BDNF, and TUNEL-positive cells than the other groups. Among OVX animals, the DW and E2 groups had fewer 5-HT2AR, BDNF, and TUNEL-positive cells than other groups. Figures 5-7 show immunological tagging of 5-HT2AR, BDNF, and TUNEL-positive cells in the hippocampus.

## DISCUSSION

In this study, we found no differences in the mean immobility times between the SHAM and OVX animals in all treatment groups except the DW groups. Especially when compared with the DW groups, the antidepressant effect of LD-LBP was clearly observed. We observed positive effects of LD-LBP and IMP on both SHAM and OVX groups. Because LD-LBP and IMP treatments reduce immobility in healthy rats (SHAM), we observed significant differences between LD-LBP and IMP treatments in both SHAM groups and OVX groups. LD-LBP and IMP showed an antidepressant effect in the menopausal depression model by increasing SOD enzyme activity and decreasing the MDA level in serum samples. Besides, they reduced cell death and increased 5-HT2AR-positive and BDNF-positive cell count in the hippocampal region.

Depression is more prevalent in women than in men (24), and in women aged 45-55 than in other age groups (25). Changing hormone profiles during menopause can cause changes in the body, increasing the risk of depression (26). The decline in estrogen levels during menopause can profoundly change brain activity, proinflammatory reactions, and oxidative stress, all of which can result in pessimism and anxiety (27). Having a bilateral ovariectomy before going through menopause naturally is linked to the onset of anxiety and depressive disorders, as well as a higher risk of cognitive decline (28).

OVX models have been created with animals of different ages and with different E2 withdrawal times after OVX (29). In our study, three-month-old rats, a 14-day recovery period, and a 30-day DW treatment period were used. At the end of the 44-day period after OVX, we observed a non-significant decrease in estrogen. Fortunately, this reduction was enough to induce depression as confirmed by reduced mobility on the FST test.

In clinical conditions, ERT is used to restore estrogen levels in women with menopausal depression (30). This treatment shows an antidepressant effect in both clinical and experimental studies (31). In our study, no significant antidepressant effect of estrogen treatment on depression-like behaviors was observed. This could be attributed to the limited sample size in the groups or insufficient E2 withdrawal time after OVX.

In this study, LBP strengthened the antioxidant system by decreasing oxidative stress caused by estrogen deficiency. Post-OVX depression was treated with low-dose LBP and IMP. The effect of LBP was similar to that of IMP, which is a clinically used antidepressant. Both LD-LBP and IMP increased the mobility time in the FST test by increasing serum SOD activity and decreasing serum MDA level to a similar degree.

While there was no significant difference in the serum levels of other antioxidant enzymes between the SHAM and OVX groups, serum GPX was significantly different: in the low-dose LPB group, GPX was higher in SHAM animals than in OVX animals, while in the high-dose LPB group it was higher in OVX animals than in SHAM animals. It was considerably reduced in exogenous estrogen groups. As a result, low-dose goji berry extract favorably affected GPX level in the SHAM group, where the estrogen level was normal, and high-dose goji berry extract favorably affected the GPX level in the OVX group, where the estrogen level was comparatively low. Since GPX regulates lipid peroxidation and is cellularly protective, goji berries can be used by both young women and those undergoing surgical menopause.

Estrogen modulates the serotonergic system in the hippocampus, one of the most important brain regions when it comes to anxiety, depression, and learning processes (32-34). Serotonergic changes that occur in estrogen deficiency may be reversed by antidepressants (35). In particular, behavioral disorders may be caused through alterations in 5-HT receptors, 5-HT1A, and 5-HT2A (36). These receptors play a role in neuronal migration (37), synapse formation (38), and neuronal proliferation (39), as well as influence behavior by modulating the serotonergic system (40). Estrogen has been shown to alter the 5-HT transporter (SERT) situated at the terminal ends of neurons, increasing the 5-HT reuptake (41). In ovariectomized monkeys, E2 modulated tryptophan hydroxylase activity, SERT, and 5-HT1AR (42). As a result, protecting the serotonergic system in this region is critical for modulating depression. The majority of antidepressants restore the lowered 5-HT level by inhibiting 5-HT reuptake (43). In our study, LBP and IMP enhanced 5-HT2AR in the hippocampus of OVX rats. HD-LBP outperformed LD-LBP and ERT. LD-LBP treatment may exert its antidepressant effects through the hippocampal mechanism as well as through the antioxidant mechanism. Other research showed the active ingredients of *Hypericum perforatum* (St John's wort) hypericin and hyperforin acting as 5-HT reuptake inhibitors in depression (14,15). Similar to IMP, LBP therapy may inhibit 5-HT reuptake by blocking SERT. Future studies should elucidate the mechanisms underlying the effect of LBP therapy on 5-HT levels.

Estrogen insufficiency inhibits hippocampal neurogenesis (44) and can lead to depression due to a decreased cell proliferation in the hippocampus area (45). The serotonergic system is strongly linked to BDNF, a protein substantially affecting hippocampal neurogenesis (46). A decreased BDNF level disrupts the survival of serotonergic neurons (46). The BDNF level decreases in depression and is reversed with antidepressants (47). In this study, the DW-OVX group had the lowest level of hippocampal BDNF. A decreased E2 level after OVX might disrupt the BDNF pathway because E2 regulates BDNF expression (44). In addition to the antioxidant impact outlined above, IMP therapy had the strongest effect on the hippocampus BDNF levels in OVX rats. HD-LBP and LD-LBP showed similar results to IMP treatments.

Estrogen is an anti-apoptotic agent with neuroprotective effects on the brain (48). After OVX in rats, the hippocampal TUNEL-positive cell count was decreased (48) and reversed by antidepressant therapy such as IMP (49). IMP provided neuroprotection against lipopolysaccharide-induced apoptosis mediated by BDNF (49). LBP treatment decreased the count of TUNEL-positive cells in retinal neurons after retinal ischemia/reperfusion injuries (50). In addition, pre-treatment LBP reduced the number of TUNEL-positive cells, preventing focal cerebral ischemia injury by reducing neuronal apoptosis in mice (51). Our results, in agreement with the literature findings, showed that after OVX, LBP and IMP decreased apoptosis in the hippocampus. That is, LBP reversed increased apoptosis in menopausal depression in a similar way as IMP. Especially, HD-LBP was a more effective treatment in the hippocampal area than LD-LBP.

Bilateral ovariectomy is a method used to assess behavioral issues following ovariectomy and the anxiolytic and antidepressant effects of herbal or pharmaceutical products (52). Ovariectomized rats have been employed in several studies on the neurological basis of anxiety and depressive symptoms linked to surgical menopause (53,54). Ovariectomy is a useful technique for assessing the effects of specific medications, but it is not a precise replica of natural menopause in humans and other mammals (27,55).

In conclusion, this study demonstrated the antidepressant effects of low and high doses of LBP. Due to its powerful antioxidant properties and positive effects on the hippocampal area, LBP can be used in addition to antidepressant treatment. Future studies should compare the effects of LBP on depression-like behavior with those of other antidepressants. Furthermore, the mechanism by which SERT or tryptophan hydroxylase activity levels increase hippocampal 5-HT should be investigated. Besides, the positive effects of HD-LBP on the hippocampal region should be investigated in learning and memory studies.
